# Application of the Fenton Process for the Removal of Emerging Contaminants in Real Wastewater—A Short Review

**DOI:** 10.3390/molecules31111916

**Published:** 2026-06-02

**Authors:** Alexis Rubén Bracamontes-Ruelas

**Affiliations:** 1 Departamento de Ingeniería Sustentable, Centro de Investigación en Materiales Avanzados, S.C., Calle CIMAV 110, Ejido Arroyo Seco 34147, Durango, Mexico; alexis.bracamontes@cimav.edu.mx; 2 Departamento de Ingeniería en Metalúrgica, Universidad Politécnica de Cuencamé, Cuencamé 35805, Durango, Mexico

**Keywords:** wastewater, emerging contaminants, homogeneous Fenton process, photo-Fenton, Fenton-like, heterogeneous Fenton, electro-Fenton, removal, treatment

## Abstract

Real wastewater contains emerging contaminants that pose problems for flora, fauna, and human health. Conventional wastewater treatment processes, such as the activated sludge process and aerated lagoons, which are commonly used worldwide, cannot remove these contaminants. Therefore, this review analyzes the application of the Fenton process and its variants—homogeneous Fenton, photo-Fenton, Fenton-like, heterogeneous Fenton, and electro-Fenton—to remove various emerging contaminants belonging to different groups, such as pharmaceuticals, personal care products, perfluoroalkyl and polyfluoroalkyl substances (PFASs), etc., from wastewater. The review focuses on the reaction mechanisms, application considerations, parameters, and future perspectives of these processes. The compiled information shows that the Fenton process and most of its variants can successfully remove emerging contaminants from different types of aqueous matrices. However, improvements are still needed in terms of performance and application for treating real wastewater on a macro scale.

## 1. Introduction

Several urban activities, such as manufacturing, agriculture, livestock, personal and health care, among others, generate significant amounts of contaminants, among which emerging contaminants (organic micropollutants or emerging pollutants) stand out [1,2].

Emerging contaminants can be produced naturally or synthetically. They are typically found in aqueous environments at low concentrations ranging from nanograms to micrograms per liter [2]. These contaminants can be classified into various groups, such as pharmaceuticals, personal care products, surfactants, hormones, flame retardants, perfluoroalkyl and polyfluoroalkyl substances (PFASs), among others [3].

Emerging contaminants are commonly used and discharged to wastewater by humans as they use them to improve their lifestyle and quality of life. As a result, humans have created over 140,000 compounds [4] and chemical compositions, from which a large number of emerging contaminants can be derived.

The main entry of emerging contaminants into the environment is through wastewater (treated and untreated) [3,5], because conventional processes commonly used (e.g., activated sludge) to treat wastewater in most parts of the world do not have the capacity to remove in their totality all of the emerging contaminants [3,5,6,7]. In addition, emerging contaminants can cause chronic negative impacts to ecosystems (animals, plants and humans) in general [3,8] and diminish the quality of any water with which they interact [8,9].

Table 1 lists some of the health problems that different emerging contaminants can cause, as well as the variability of their concentrations in wastewater.

Therefore, processes are needed that have the capacity to remove (general term used throughout the text to refer to the mineralization, oxidation or degradation of emerging contaminants) emerging contaminants from wastewater, either as primary processes or as coupled processes (tertiary or quaternary processes) to existing conventional WWTPs.

In response, researchers have widely recommended advanced oxidation processes as a solution to the aforementioned problem due to that advanced oxidation processes have the ability to remove organic contaminants (emerging contaminants) from wastewater completely or partially and not only concentrate or phase shift them like other processes (e.g., flocculation, coagulation, and membranes) [9,11].

Advanced oxidation processes can be generally defined as all those methods that generate hydroxyl radicals (HO^•^, oxidative species that remove or oxidize organic matter contamination from wastewater) in situ [12]. Hydroxyl radicals (HO^•^) are non-selective radicals [13] and have a high oxidative power amounting to 2.8 eV [5,14].

Typically, catalytic agents (e.g., Fe^2+^), oxidizing agents (e.g., H_2_O_2_), and sometimes even energies external to the process such as solar radiation in combination are used to carry out the generation of hydroxyl radicals (HO^•^) in advanced oxidation processes [5,9]. Advanced oxidation processes are normally classified as chemical, electrochemical, and photochemical, among others, depending on the chemical agents (catalytic and oxidizing) and energy sources used as precursors for the generation of hydroxyl radicals (HO^•^) [15].

Common advanced oxidation processes include Fenton, photo-peroxidation, indirect ozonation, photocatalysis, and so on [9,16,17].

However, among the advanced oxidation processes, the Fenton process has been widely applied due to its low cost and ease of use [18]. Also, the Fenton process has become one of the best options to remove difficult-to-treat organic contaminants (e.g., emerging contaminants) from wastewater [19] and has shown effectiveness in removing recalcitrant contaminants (emerging contaminants) as a single or coupled process with conventional wastewater treatments [20].

Given such perspectives, the present document aims to address the most relevant information (application considerations, parameters, reaction mechanisms, and perspectives, among others) about the Fenton process and its variants (homogeneous Fenton process, photo-Fenton, Fenton-like, heterogeneous Fenton, and electro-Fenton) for the removal of emerging contaminants in real wastewater. In addition, the document shows that the Fenton process and its variants need to be applied on a large scale; finally, it highlights the need to transition to testing in actual wastewater rather than synthetic water. This makes it a timely and valuable review for the field.

Nevertheless, this manuscript serves as a reference framework for some researchers, as it systematically details the reaction mechanisms; critical operating parameters; and the essential steps for the practical implementation of the Fenton process and some of its variants.

## 2. Fenton Process

As the author has already noted in previous studies the Fenton process was discovered by Henry John Horstman Fenton in 1894, when he observed that hydrogen peroxide (H_2_O_2_) and iron salts oxidized tartaric acid in an acidic aqueous solution [5,21]. The Fenton process is encompassed by the reaction mechanisms shown in Equations (1) and (2) [5,9,20,22].

In Equation (1) the catalyst (Fe^2+^) reacts with the hydrogen peroxide (H_2_O_2_), producing hydroxyl radicals (HO^•^), which are the oxidative species responsible in this process for the removal of emerging contaminants from wastewater [22].

(1) $${Fe}^{2+}+H_{2}O_{2}\to {Fe}^{3+}+HO^{-}+HO^{•}$$

Additionally, if there is hydrogen peroxide (H_2_O_2_) remaining in the wastewater, by means of the reaction mechanism shown in Equation (2), hydrogen peroxide (H_2_O_2_) can subsequently react with the ferric ions (Fe^3+^) generated as a by-product in Equation (1) and produce other oxidative species, such as perhydroxyl radicals (HO_2_^•^) [22]. Perhydroxyl radicals (HO_2_^•^) have a lower oxidative power than hydroxyl radicals (HO^•^) [5], but like hydroxyl radicals (HO^•^), they can contribute to the removal of emerging contaminants in wastewater.

(2) $${Fe}^{3+}+H_{2}O_{2}\to {Fe}^{2+}+H^{+}+HO_{2}^{•}$$

The Fenton process described chemically in a general way by Equations (1) and (2) is known as the homogeneous Fenton process, because this type of process is carried out in the entire aqueous phase of the wastewater to be treated.

**Figure 1:**
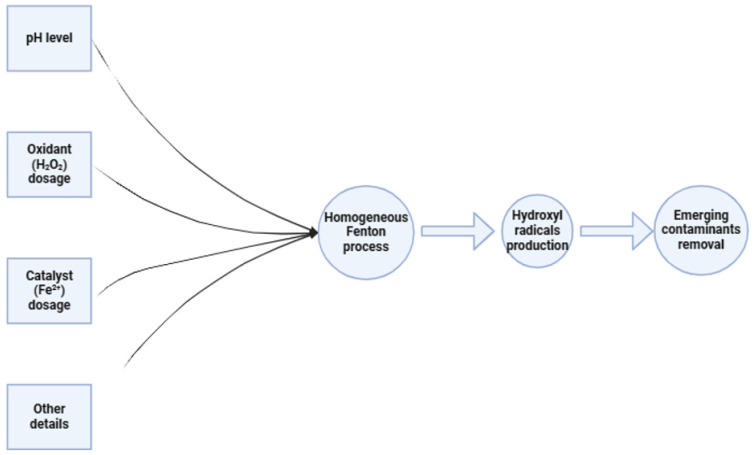
Important operational criteria (parameters) in the homogeneous Fenton process for the removal of emerging contaminants from wastewater.

It should be remarked that for the homogeneous Fenton process to produce hydroxyl radicals (HO^•^) and remove the emerging contaminants from wastewater, certain operating criteria such as pH, oxidant dosage (H_2_O_2_), catalyst dosage (Fe^2+^), and other details must be considered (Figure 1).

### 2.1. pH

In the homogeneous Fenton process, to produce hydroxyl radicals (HO^•^) and remove the emerging contaminants of interest, a pH level around of 3 in the wastewater must be managed [5,9,23,24]. Because the homogeneous Fenton process is directly pH-dependent, if the pH increases to neutral ranges, iron hydroxide precipitates are generated, and if the pH decreases below 3, complex iron species are generated; this affects the production of hydroxyl radicals (HO^•^), since in both cases the free iron species are lower (e.g., Fe^2+^) [5,23].

Furthermore, it is necessary to avoid adjusting the pH of the wastewater to be treated by the homogeneous Fenton process with hydrochloric acid (HCl) [9], since the chloride (Cl^−^) obtained when such acid is dissociated in the wastewater can act as a scavenger of hydroxyl radicals (HO^•^) and therefore can reduce the removal performance of the emerging contaminants of interest in the wastewater treated by the homogeneous Fenton process [9,25].

Finally, it should be noted that pH can affect the removal of emerging contaminants during the Fenton process because it influences the speciation and ionization of contaminants. Therefore, the negative logarithm of the acidity constant (pK_a_) must be considered because it can indicate the stability of an emerging contaminant and its ability to interact with hydroxyl radicals (HO^•^) [26]. Additionally, hydrogen peroxide (H_2_O_2_) has been found to be more stable under acidic conditions [27].

### 2.2. Oxidant Dosage (H_2_O_2_)

Regarding the dosage of the oxidant (H_2_O_2_), it is well established that it is a crucial parameter in carrying out the homogenous Fenton process and removing emerging contaminants from wastewater, given that if the oxidant (H_2_O_2_) is added in considerable quantities, the excess of oxidant (H_2_O_2_) can cause problems with the process, such as increased costs and the propagation of secondary scavenger reactions, which affect the generation of hydroxyl radicals (HO^•^), as shown in Equations (3) and (4) [28,29].

(3) $$H_{2}O_{2}+HO^{•}\to H_{2}O+HO_{2}^{•}$$

(4) $$HO_{2}^{•}+HO^{•}\to H_{2}O+O_{2}$$

Several researchers have proposed that in order to establish the dosage of the oxidant (H_2_O_2_) for the removal of emerging contaminants in the wastewater to be treated, each case must be examined [28] and therefore evaluated at the laboratory level to make an adequate oxidant (H_2_O_2_) dosage.

Making the oxidant (H_2_O_2_) dosages previously at the laboratory level has very little applicability in reality for the treatment of wastewater in a full scale-up for the simple fact that if a statistical optimization model is made at the laboratory level for the treatment of a certain type of wastewater and the removal of emerging contaminants, it would only be applicable for that particular case. On the other hand, time would be wasted in doing the laboratory experiments beforehand to determine the dosages of the oxidant (H_2_O_2_) to be added to the wastewater to remove the emerging contaminants of interest. So, having said that, as the author has already noted in previous studies researchers have widely applied Equation (5) to dose the oxidant (H_2_O_2_) and solve such problems [5,9,30,31].

(5) $$Quantity\;or\;dosage\left(mg\right)\;H_{2}O_{2}=\frac{17}{8}\left[COD,mg\cdot L^{-1}\right]\times V(L)$$

In Equation (5), the oxidant (H_2_O_2_) dosage is calculated as a function of the initial total chemical oxygen demand (COD) and the volume (V) of the wastewater to be treated. The initial COD is entered into Equation (5) in mg·L^−1^ in addition to the volume (V) of wastewater to be treated in liters (L).

It should be noted that the oxidant (H_2_O_2_) dosages in Equation (5) are calculated with respect to the initial total COD of the wastewater to be treated, because if this dosage was calculated only in terms of the emerging contaminant concentrations of interest to be removed from wastewater, since hydroxyl radicals (HO^•^) are non-selective oxidative species, this dosage could be underestimated, owing to that the real wastewater does not only contain emerging contaminants as organic contaminants [5,9].

### 2.3. Catalyst Dosage and Type

For its part, the catalyst (Fe^2+^), if added in excessive amounts, can also cause problems in the hydroxyl radical (HO^•^) generation of the homogeneous Fenton process, as shown in Equation (6), for the reason that excessive catalyst (Fe^2+^) can react with the hydroxyl radicals (HO^•^) and generate other undesirable species (Equation (6)) in the process. It can thus decrease the emerging contaminant removal efficiency of the homogeneous Fenton process [29].

(6) $$Fe^{2+}+HO^{•}+H^{+}\to Fe^{3+}+H_{2}O$$

In order to solve the problem of catalyst (Fe^2+^) dosing, some researchers have taken advantage of the 1:1 ratio between the catalyst (Fe^2+^) and the oxidant (H_2_O_2_) established by the Fenton reaction (Equation (1)) and have used Equation (7) for the catalyst (Fe^2+^) dosage to remove emerging contaminants from wastewater [5,9].

As the author has already noted in previous studies in Equation (7), nmol Fe^2+^ are the moles of catalyst (Fe^2+^) calculated by the 1:1 ratio of oxidant (H_2_O_2_) to catalyst (Fe^2+^) in the Fenton reaction (Equation (1)), and 278,010 is the molecular weight in milligrams of heptahydrated ferrous sulfate (FeSO_4_•7H_2_O) [9,31].

(7) $$Quantity\;or\;dosage\left(mg\right)\;FeSO_{4}•7H_{2}O=(278,010)(nmol\;{Fe}^{2+})$$

Note that Equation (7) calculates the dosage of heptahydrated ferrous sulfate (FeSO_4_•7H_2_O), because, in wastewater treatments, it is the reagent supplier of the catalyst (Fe^2+^) used for excellence in the homogeneous Fenton process in several investigations [9,32].

Finally, as a suggestion, Equations (1), (5) and (7) can be used together for the dosing of the oxidant (H_2_O_2_) and catalyst (Fe^2+^) as a function of the initial total COD that presents the wastewater to be treated in the homogeneous Fenton process for the removal of the emerging contaminants of interest. Nonetheless, it is at the discretion of the researchers, as some researchers have proposed in the case of the oxidant (H_2_O_2_) dosage in the homogeneous Fenton process that the catalyst (Fe^2+^) dosages should be previously examined and proposed at the laboratory level for each case in particular.

### 2.4. Performance of the Homogeneous Fenton Process

**Figure 2:**
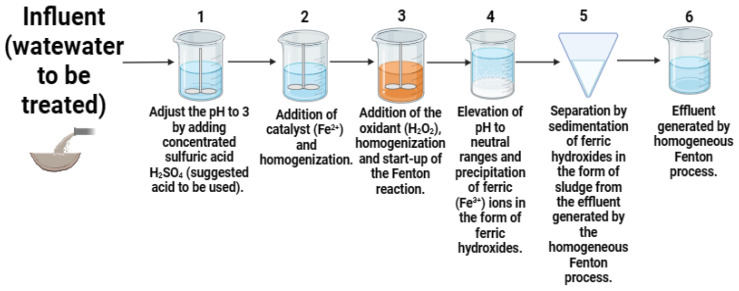
Steps to perform the homogeneous Fenton process.

Now, taking into account what was described in the previous subsections, in order to carry out the homogeneous Fenton process and remove the emerging contaminants from the wastewater, the steps shown in Figure 2 must be performed.

In the homogeneous Fenton process (Figure 2), first, to perform it and remove the emerging contaminants from the wastewater, the pH (step 1) of the wastewater needs to be adjusted to 3. After that, the catalyst (Fe^2+^) must be added (step 2), and finally, the oxidant (H_2_O_2_) duty needs to be added (step 3). In step 3 (Figure 2), the wastewater is dyed orange, because when the homogeneous Fenton process is carried out, in addition to producing hydroxyl radicals (HO^•^) (oxidative species of interest), ferric (Fe^3+^) ions are also generated as a by-product (Equation (1)). Ferric ions (Fe^3+^) give that characteristic color to the wastewater when the Fenton reaction is carried out. Nevertheless, in order to remove the characteristic orange color of the wastewater treated by the homogeneous Fenton process, the ferric (Fe^3+^) ions must be removed, in the form of ferric hydroxides (Fe(OH)_3_), by raising the pH to neutral ranges (pH = 7), as shown in step 4 of Figure 2 [33].

To end the homogeneous Fenton process, in step 5, by means of simple sedimentation processes (Figure 2), the ferric hydroxides (Fe(OH)_3_) generated in the homogeneous Fenton process are removed from the treated wastewater in the form of sludge, and the treated wastewater is obtained in step 6 (Figure 2) [33].

It is worth mentioning that in the homogeneous Fenton process, to adjust the pH to acidic (step 1) and neutral (step 4) ranges from the wastewater to be treated, sulfuric acid (H_2_SO_4_) and sodium hydroxide (NaOH) are generally used in most of the investigations [28]. But the type of acid and base to be used for pH adjustments in the homogeneous Fenton process is left to the researcher’s consideration.

### 2.5. Removal of Emerging Contaminants in Wastewater by the Homogeneous Fenton Process

The homogeneous Fenton process was applied for the removal of emerging contaminants in different real wastewaters as a unique or coupled treatment (Table 2). Researchers such as Guo et al. [34] and Ishak and Malakahmad [35] have shown the good applicability of the homogeneous Fenton process as a unique process at the laboratory level for the treatment of different wastewater. Guo et al. [34], using a pH = 4.13, an oxidant concentration (H_2_O_2_) of 1 mol·L^−1^ and a catalyst (Fe^2+^) concentration of 0.36 mol·L^−1^, obtained a removal percentage of benzene dye intermediates in treated wastewater of 85.29% (Table 2). Furthermore, the color of the real treated wastewater was removed in a percentage of 99.99%, and it was shown, according to analyses carried out, that the effluent generated by the homogeneous Fenton process was not toxic [34].

For their part, Ishak and Malakahmad [35] treated real wastewater from an oil refinery at the laboratory level by means of the homogeneous Fenton process using molar ratios of 2.8 H_2_O_2_/COD, 4 H_2_O_2_/Fe^2+^ and a pH = 3 and found that the partial oxidation of the refinery compounds by the homogeneous Fenton process reduced the toxicity of the treated wastewater in 71 min of hydraulic retention. Moreover, they identified that more than 90% of the COD (Table 2) was removed in the first 20 min of hydraulic retention, indicating the feasibility of using this process as unique for the treatment of this type of wastewater [35].

On the other hand, Bracamontes-Ruelas et al. [31] and López-Velázquez et al. [36] used the homogeneous Fenton process as a process coupled (Table 2) with other wastewater treatments for the removal of emerging contaminants, showing good results.

Research by Bracamontes-Ruelas et al. [31] showed that the homogeneous Fenton process was able to simultaneously remove emerging contaminants such as triclosan, ibuprofen, carbamazepine, caffeine, acesulfame-K and DEET in wastewater from a secondary process of a conventional activated sludge WWTP, obtaining removal percentages, except for DEET (85.21%), of almost 100% (Table 2) for all emerging contaminants in 60 min [31]. Similarly, in a study by López-Velázquez et al. [36], emerging contaminants such as 17α-ethinylestradiol and caffeine were also removed at percentages greater than 99% (Table 2) from wastewater from a UASB (up-flow anaerobic sludge blanket reactor) treatment in 60 min.

It is important to note that in all the investigations shown in Table 2, ferrous sulfate heptahydrate (FeSO_4_•7H_2_O) was used as the catalyst (Fe^2+^) supplier compound, and sulfuric acid (H_2_SO_4_) and sodium hydroxide (NaOH) were used to adjust the pH of the wastewater to be treated. In addition, in most cases, the investigations (Table 2) for the removal of emerging contaminants by means of the homogeneous Fenton process used a pH = 3 and an approximate treatment time of 60 min, and the catalyst (Fe^2+^), and the oxidant (H_2_O_2_) dosages varied in function of the type of wastewater to be treated and the type of emerging contaminants to be removed by the homogeneous Fenton process.

As final part, to conclude, the research compiled and described in this subsection (Table 2), as is well known, evidence and highlight the feasibility of the homogeneous Fenton process as a unique or coupled treatment for the removal of emerging contaminants in different types of wastewater (Table 2). Moreover, it has been shown that perfluoroalkyl and polyfluoroalkyl substances (PFASs), which are persistent emerging contaminants that pose a significant threat to the environment and human health due to their chemical stability and potential for bioaccumulation, can be removed by the homogeneous Fenton process, as this method offers several advantages, including mild reaction conditions, operational simplicity, and cost-effectiveness [37].

## 3. Problems in the Application of the Homogeneous Fenton Process and Its Variants

**Figure 3:**
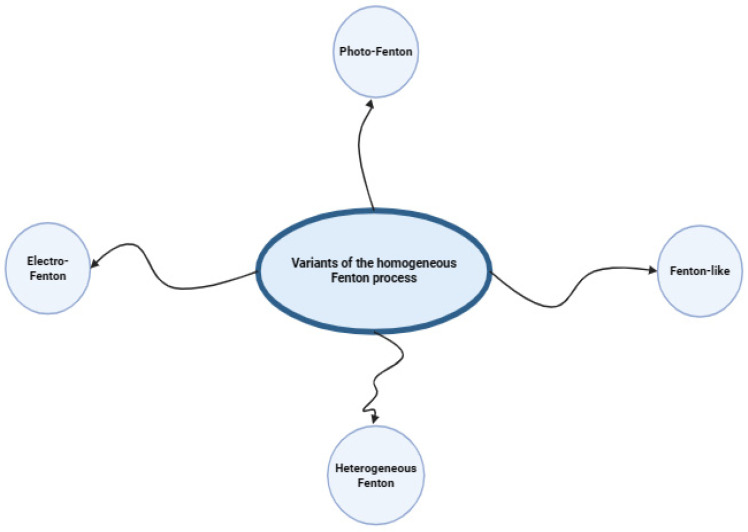
Variants of the homogeneous Fenton process.

Nonetheless, although the homogeneous Fenton process efficiently removes the emerging contaminants in different types of wastewater, as shown in Table 2, the process presents some catalyst (Fe^2+^)-related problems and challenges, among which are the generation of sludge (iron ions precipitated in the form of iron hydroxides upon raising the pH), the loss of iron ions, and the adjustment of the pH acid level of 3 required to carry out the Fenton reaction (additional costs generated by the process to adjust the pH) [5,38,39,40]. Thus, for these reasons, researchers have developed and proposed processes (variants of the homogeneous Fenton process) such as photo-Fenton, Fenton-like, heterogeneous Fenton and electro-Fenton to solve the aforementioned problems (Figure 3) [38].

### 3.1. Photo-Fenton

The photo-Fenton process is a variant of the homogeneous Fenton process, where light energy (UV, visible or sunlight) is added to solve some limitations (reuse of ferric (Fe^3+^) ion generated by-product in Equation (1)) of the normal homogeneous Fenton process. In this process the efficiency of the Fenton reaction is increased by the photo reduction of the ferric ions (Fe^3+^) generated as a by-product in the classical Fenton reaction (Equation (1)) to ferrous ions (Fe^2+^), as shown in Equation (8) [41,42]. For the reaction (Equation (8)) of the photo-Fenton process to take place and generate hydroxyl radicals (HO^•^), it should be mentioned that wavelengths of light energy in the process of less than 600 nanometers (λ<600 nm) must be handled [41].

(8) $$Fe^{3+}+H_{2}O+hv\to Fe^{2+}+HO^{•}$$

It should be noted that the photo-Fenton process can further enhance the removal of emerging contaminants in the wastewater compared to the homogeneous Fenton process, since the process generates hydroxyl radicals (HO^•^) by means of Equations (1) and (8). In addition, if the wavelength of the light energy used is less than 310 nanometers (λ<310 nm), the photo-peroxidation process can be carried out simultaneously with the photo-Fenton process by means of Equation (9), which would enhance the production of hydroxyl radicals (HO^•^) in the process significantly and thus increase the removal of emerging contaminants from the wastewater [42,43].

This indicates, in summary, that the photo-Fenton process has three more alternative routes to generate hydroxyl radicals (HO^•^) than the homogeneous Fenton process, the first being the photo reduction (Equation (8)) of the ferric ions (Fe^3+^) generated in the Fenton reaction, the second being the possible interaction of the ferrous ions (Fe^2+^, product of the photo reduction) with remanent hydrogen peroxide (H_2_O_2_) if it exists in the wastewater, and the third (Equation (9)) being the generation of hydroxyl radicals (HO^•^) by means of the photolysis of hydrogen peroxide (H_2_O_2_) if a wavelength lower than 310 nanometers is managed [43].

(9) $$H_{2}O_{2}+hv\to 2HO^{•}$$

As an additional important fact, it must be said that when a UVC lamp (λ=252.7 nm) is used to carry out the photo-Fenton process, the photo-Fenton reaction (Equation (8)) is not the predominant one, but the photo-peroxidation reaction is (Equation (9)) [42,44].

Furthermore, according to research, in order to carry out the photo-Fenton process and remove the emerging contaminants of interest from the wastewater, a pH of around 3 as in the homogeneous Fenton process must be managed, and the molar ratios of the catalyst (Fe^2+^) and oxidant (H_2_O_2_) for their dosage must be 2 H_2_O_2_/Fe^2+^ and 1–3.5 H_2_O_2_/COD, depending on the type of water and emerging contaminants to be removed in each case particular [45].

Nonetheless, the dosages of the catalyst (Fe^2+^) and the oxidant (H_2_O_2_), as in the homogeneous Fenton process, can also be calculated by means of Equations (1), (5) and (7), since ferrous sulfate heptahydrate (FeSO_4_•7H_2_O) is the catalyst (Fe^2+^) supplier reagent usually used in the photo-Fenton process, but this will be left to the decision of the researcher [9].

Having said this, the photo-Fenton process can be carried out in the same way as the traditional homogeneous Fenton process; as shown in Figure 2, only an additional light energy source would have to be added.

Now, although the photo-Fenton process presents some improvements compared to the homogeneous Fenton process and has been considered by many researchers as a viable method to remove emerging contaminants (e.g., pharmaceuticals) from wastewater (Table 3) [46,47,48,49,50,51], this process also has some disadvantages to be highlighted, such as the pH adjustment to approximately 3 in the treated water that has to be managed to keep the iron ions in their soluble form and the pH elevation of the treated wastewater that has to be raised to 7 in order to discharge it into the environment [47].

Additionally, in comparison to the homogeneous Fenton process, the photo-Fenton process presents two more operational problems related to illumination, with the first one being that if the wastewater is not irradiated in an adequate way by the light source used, the removal of the target emerging contaminants may decrease. On the other hand, if the wastewater to be treated has a high turbidity, the propagation of the light energy in the wastewater might be affected, and therefore it would decrease the removal of the emerging contaminants of interest from the wastewater [9,50].

Consequently, given the information shown in Table 3 and compiled in this subsection, it can be said that the photo-Fenton process, like its predecessor (homogeneous Fenton process), has a good capacity to treat different types of wastewater and remove emerging contaminants as a single or coupled process (Table 3).

However, it presents more operational problems than the homogeneous Fenton process when adding light radiation for the reuse of the ferric (Fe^3+^) ion (photoreduction) generated by-product in Equation (1), due to the simple fact that the turbidity of the wastewater can affect the removal performance of the emerging contaminants of the process and the radiation must be widely distributed throughout the aqueous medium to carry out the photo-Fenton process. This is in addition to that the pH must be adjusted as in the traditional homogeneous Fenton process (Figure 2).

Finally, it should be mentioned that if it is desired to use solar energy as a light source in the photo-Fenton process for the removal of emerging contaminants from the wastewater to be treated, translucent materials in the reactor must be used in order to maximize the use of solar energy to remove the emerging contaminants of interest [9].

### 3.2. Fenton-like

The Fenton-like process used to treat wastewater and remove emerging contaminants is so named because, in comparison to the homogeneous Fenton process, this process uses other metals (e.g., Cu^+^, Mn^2+^ and Co^2+^) instead of the ferrous (Fe^2+^) ion as catalysts to initiate the Fenton reaction and generate hydroxyl radicals (HO^•^) [19,52,53].

An example of the Fenton-like reaction using another metal as the catalyst is given by Equation (10). It should be mentioned that the normally substituted catalyst of the ferrous ion (Fe^2+^) in the Fenton-like process has the lowest atomic oxidation states of its species [19].

(10) $$Cu^{+}+H_{2}O_{2}\to Cu^{2+}+HO^{-}+HO^{•}$$

It is commonly recommended that metals with a lower oxidation state of their species be used in the Fenton-like process, since if a metal with a higher oxidation state is used, perhydroxyl radicals (HO_2_^•^) could be generated as in Equation (2) of the traditional Fenton process. Therefore, the removal capability of the Fenton-like process of emerging contaminants would be reduced, due to the fact that, as mentioned above, pehydroxyl radicals (HO_2_^•^) have a lower oxidative power than hydroxyl radicals (HO^•^). Furthermore, the fact that perhydroxyl radicals (HO_2_^•^) are generated when a metal with the highest oxidation state is used in the Fenton-like process instead of the metal with the lowest oxidation state is due to the fact that instead of oxidation of the metal in the chemical reaction, a reduction is carried out, as exemplified in Equations (1) and (2), which are the traditional reactions of the homogeneous Fenton process. On the other hand, in the Fenton-like process, as in the traditional homogeneous Fenton process, the oxidant (H_2_O_2_) par excellence is hydrogen peroxide (H_2_O_2_), and likewise, a pH of approximately 3 must be managed [54,55,56].

At the same time, the dosages of both the catalyst (metal to be used) and the oxidant (H_2_O_2_) must be previously considered by performing laboratory tests to carry out the removal of the emerging contaminants in the wastewater, since if they are dosed excessively, both the catalyst and the oxidant (H_2_O_2_) of the Fenton-like process could have scavenging effects of hydroxyl radicals (HO^•^) and therefore decrease the removal performance of the emerging contaminants of interest. Now, similarly, if the catalyst and oxidant (H_2_O_2_) are dosed in amounts less than those required for the removal of the emerging contaminants of interest from the wastewater to be treated, the emerging contaminant removal performance provided by the Fenton-like process would also be affected [56,57]. Therefore, it is recommended that the dosages of the catalyst and oxidant (H_2_O_2_) in the Fenton-like process be carefully examined and defined by the researcher in each case in particular.

It is advisable to say that the type of catalyst to be used in the Fenton-like process for the removal of the emerging contaminants of interest will be left to the decision of the researcher, because it can influence the mode of operation and costs of the process, and additionally, it can generate secondary potential contamination [58].

Nonetheless, it should be emphasized that although the Fenton-like process has shown the ability to use different catalysts in several investigations for the removal of both emerging contaminants and common contaminants, improvements in the process are still lacking, and the applicability of the process in real wastewater to remove emerging contaminants is questionable because the investigations to evaluate this process are commonly done in synthetic water [55,59,60,61]. On the other hand, it is necessary to emphasize that in the Fenton-like process several types of catalysts have been synthesized to solve the problems presented by the traditional homogeneous Fenton process [61]. However, there is a need to evaluate how much it costs to synthesize this type of catalyst, since it might be a solution to a problem presented by the traditional Fenton process but it would be incurring economic problems, which could hinder the applicability of the process at a macro level.

**Figure 4:**
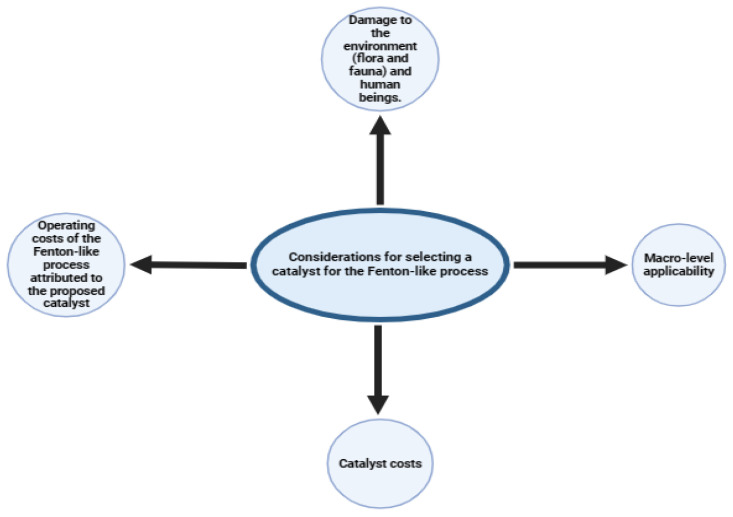
Considerations for the selection of the catalyst to be used in the Fenton-like process.

To conclude, given the perspectives mentioned above in this subsection, the Fenton-like process and its diverse use of catalysts show great progress in the field of the generation of new catalysts for wastewater treatment and removal of emerging contaminants, but it is still necessary to verify the damage they can cause to the environment, their applicability at a macro level of treatment, the costs of their elaboration and the operating costs for each type of new catalyst, as shown in Figure 4.

### 3.3. Heterogeneous Fenton

**Figure 5:**
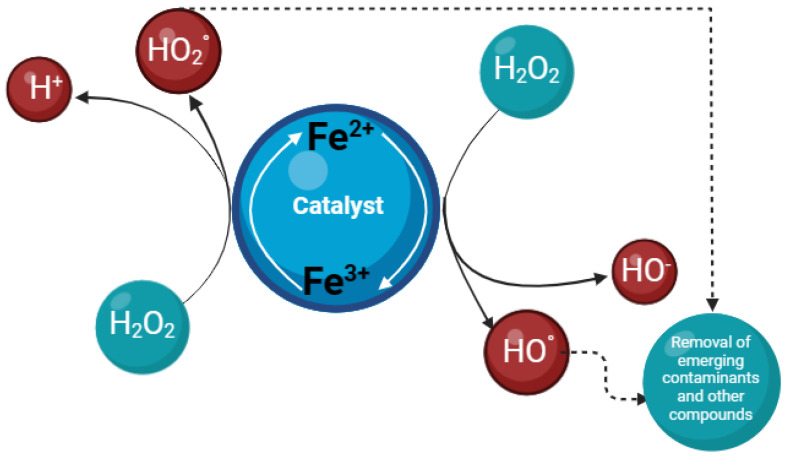
Heterogeneous Fenton reaction mechanisms for the removal of emerging contaminants in wastewater, adapted and modified from [19,28].

The heterogeneous Fenton process arises from the need to solve the problems associated with the catalyst present in the homogeneous Fenton process (Section 3), the photo-Fenton process and the Fenton-like process. In this process, the modification consists of using solid catalysts (Fe_2_O_3_, Fe_3_O_4_, FeO, and FeOOH, among others) to react with the oxidant (H_2_O_2_) and remove the emerging contaminants of interest from the wastewater by generating hydroxyl radicals (HO^•^) and perhydroxyl radicals (HO_2_^•^) (Figure 5) [62].

It is worth mentioning that in heterogeneous catalysis the iron is stabilized in the catalyst structure to generate hydroxyl radicals (HO^•^) and perhydroxyl radicals (HO_2_^•^), as shown in Figure 5 [63].

In the heterogeneous Fenton process, two reaction mechanisms are present to generate radicals (HO^•^ and HO_2_^•^) and remove emerging contaminants of interest: the first reaction mechanism is the interaction of the catalyst leachate with the oxidant (H_2_O_2_), and the second reaction mechanism is through the reaction of ferrous (Fe^2+^) ions and ferric (Fe^3+^) ions found on the surface of the solid catalyst with the oxidant (H_2_O_2_), as exemplified in Figure 5 [23,63].

Nonetheless, although the reaction mechanisms of the heterogeneous Fenton process shown in Figure 5 are widely accepted by the scientific community [18,27], there are still unresolved issues regarding the understanding of these (Figure 5) [64].

On the other hand, the compiled research shown in Table 4 has shown that various catalysts have been developed in the heterogeneous Fenton process for the removal of emerging contaminants and good results have been obtained [65,66,67].

The only problem is that they have only been tested in synthetic waters (Table 4) with a certain amount and type of emerging contaminant, which makes these good results far from reality. This shows that it is necessary to test these catalysts (Table 4) in conjunction with the heterogeneous Fenton process in real wastewater to verify their capacity to remove emerging contaminants.

It should also be noted that the heterogeneous Fenton process, according to the research compiled in Table 4, can be taken to neutral pH ranges due to the nature of the catalyst, which solves the problem present in its predecessors (homogeneous Fenton process, photo-Fenton and Fenton-like) [66,67].

However, the heterogeneous Fenton process still presents some problems in its development, since it has recently been shown that although some problems (Section 3) presented by its predecessors (homogeneous Fenton process, photo-Fenton and Fenton-like) are solved by the use of solid catalysts, other problems arise such as the affectation in the reaction kinetics [68], the limitations in mass transfer [69], the lack of applicability of the heterogeneous Fenton process for treatment of real wastewaters at a macro level, the extensive experimentation at the laboratory level to define the quantities of solid catalyst to be used, and the toxicity that this type of catalyst can cause [70]. This indicates that the heterogeneous Fenton process presents improvements through the use of solid catalysts compared to predecessors (homogeneous Fenton process, photo-Fenton, and Fenton-like), but there is still work to be done in its development. It should be noted that, at present, efforts in the heterogeneous Fenton process have focused on the use and development of high-performance single-atom catalysts, as it has been found that single-atom iron catalysts remove antibiotics with near-zero metal leaching [71].

### 3.4. Electro-Fenton

Currently, the electro-Fenton process has become an important alternative treatment for the removal of recalcitrant organic contaminants (e.g., emerging contaminants) in wastewater [72].

The principal modification of the electro-Fenton process compared to its predecessors is that the oxidant (H_2_O_2_) is electrogenerated in situ by means of the reduction of oxygen (O_2_) at the cathode (Equation (1)) [73]. It is necessary to manage an acid pH (pH = 3) in the wastewater to be treated because the electro-Fenton process generates the oxidant (H_2_O_2_) in situ through the reduction of oxygen (O_2_), as shown in Equation (11) [73,74].

(11) $$O_{2}+2H^{+}+2e^{-}\to H_{2}O_{2}$$

Additionally, it should be taken into account that in order to carry out the electrogeneration of hydrogen peroxide (H_2_O_2_) through the reduction of molecular oxygen (O_2_) in the electro-Fenton process, a potential of E^0^ = 0.695 vs. SHE should not be exceeded, because if it is exceeded, secondary reactions can be generated (Equations (12) and (13)), which can decrease the production of the oxidant (H_2_O_2_) and would affect the removal performance of emerging contaminants from the process in the wastewater categorically. It should be noted that the presence of iron ions (Fe^2+^ and Fe^3+^) in the wastewater favors the electrogeneration of the oxidant (H_2_O_2_) in this process by means of Equation (11) [75].

(12) $$H_{2}O_{2}+2H^{+}+2e^{-}\to 2H_{2}O\;\;\;\;\;E^{0}=1.776\;\mathrm{vs.}\;\mathrm{SHE}$$

(13) $$O_{2}+4H^{+}+4e^{-}\to 2H_{2}O\;\;\;\;\;\;\;\;E^{0}=1.23\;\mathrm{vs.}\;\mathrm{SHE}$$

Another important factor that must be considered for the electro-Fenton process to be carried out is that the ferrous ion (Fe^2+^) must be added to the wastewater to be treated in order for the Fenton reaction to occur (Equation (1)) and the hydroxyl radicals (HO^•^) to be generated [76]. It should be remarked that in this process the ferric ions (Fe^3+^) generated as a by-product of the Fenton reaction (Equation (1)) are also electroreduced to ferrous ions (Fe^2+^) again (Equation (14)) [77].

(14) $${Fe}^{3+}+e^{-}\to {Fe}^{2+}$$

**Figure 6:**
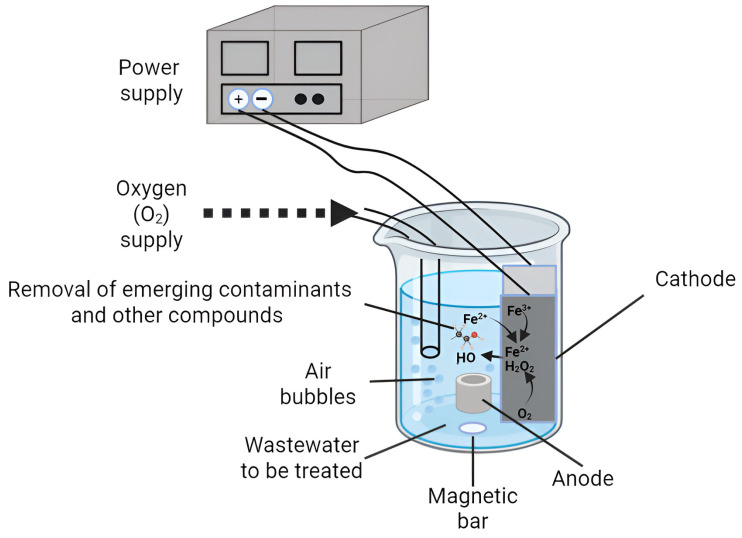
Example of experimental setup of the electro-Fenton process at the laboratory level for wastewater treatment, adapted and modified from [78].

In the electro-Fenton process two compartment cells (Figure 6) are commonly used under potentiostatic or galvanostatic conditions, and it is recommended to use a carbonous cathode and an anode with high or low oxidation power [76].

In general, Figure 6 illustrates an arrangement of the compartmental cells and how the electro-Fenton process should be carried out for the removal of emerging contaminants of interest from wastewater at the laboratory level [78]. Note, in addition, that the experimental configurations shown in Figure 2 and Figure 6 must be carried out together to perform the electro-Fenton process.

Now, it is of great importance to mention that in several investigations, it has been widely recommended that the most important parameters (operational conditions) to consider in order to carry out the electro-Fenton process and eliminate emerging contaminants from wastewater are: the design of the electro-Fenton reactor (Figure 6), the pH level (pH = 3) of the wastewater to be treated, the applied potential or current density (*j*), and the H_2_O_2_/Fe^2+^ concentration rate [79].

Notwithstanding, the reactor design, as well as the operating conditions (parameters) of the electro-Fenton process, will depend directly on the type of emerging contaminants to be removed, the type of wastewater where they are found, and the operating restrictions (e.g., pH = 3 and not to exceed E^0^ = 0.695 vs. SHE) of the electro-Fenton process, as evidenced in Table 5. Therefore, it will be necessary beforehand to perform tests at the laboratory level.

Finally, the electro-Fenton process shows a good removal capacity of emerging contaminants in different types of wastewater (aqueous matrices), according to Table 5, as a single (primary) or coupled (e.g., tertiary) treatment [81,82]. However, it still presents problems such as high treatment times for the removal of emerging contaminants, scaling-up problems, electrode passivation, elevated chemical costs for pH adjustment to acid and neutral ranges of the wastewater, considerable power consumption, sludge generation (Fe(OH)_3_), and low oxidant (H_2_O_2_) productivity attributed to low oxygen (O_2_) solubility in regular ambient conditions [83,84,85,86].

## 4. Recommendations, Considerations and Future Perspectives of the Fenton Process and Its Variants for the Removal of Emerging Contaminants in Wastewater

As a first recommendation, to carry out any variant of the homogeneous Fenton process (photo-Fenton, Fenton-like, heterogeneous Fenton, and electro-Fenton) it is suggested to base the approach on Figure 2, since these steps are general and fundamental to carry out such processes and since only changes are made depending on the catalyst (Fenton-like and heterogeneous Fenton) and the energy sources added (photo-Fenton and electro-Fenton).

Furthermore, in all variants of the Fenton process for treating wastewater and removing emerging contaminants, the amounts of the oxidant (H_2_O_2_) and the catalyst can be theoretically defined as a function of the total chemical oxygen demand (COD) that presents wastewater to be treated by means of Equations (1) and (5) [9]. It should be noted that, as in the case of the Fenton-like and heterogeneous Fenton process the type of catalysts changes, additional chemometric calculations must be performed.

On the other hand, it should be recommended that the dosages of the oxidant (H_2_O_2_) and the catalyst should be made according to the total COD or TOC (total organic carbon), because if they are made only on the basis of the concentrations of the emerging contaminants that need to be removed, these quantities would be underestimated since the hydroxyl radicals (HO^•^) are non-selective and would indiscriminately attack other types of organic contaminants contained in the wastewater to be treated; this is due to the simple fact that in real wastewater there is a higher presence of other types of organic contaminants [5,9]. Additionally, it is necessary to take into account that in order to carry out any type of Fenton process, the wastewater to be treated must have low concentrations of chlorides (Cl^−^), since these species can act as scavengers of hydroxyl radicals (HO^•^) and therefore reduce the removal performance of the emerging contaminants of interest in the wastewater [9,25].

It is necessary to say that when adding external energies (photo-Fenton and electro-Fenton) to the traditional homogeneous Fenton process and making changes to improve it (Fenton-like and heterogeneous Fenton), the complexity of operation and the difficulty of its applicability increase, as in the case of electro-Fenton (Table 6) [5]. For this reason, in the present manuscript the topics of photo-sound-electro-Fenton, heterogeneous electro-Fenton, and heterogeneous photo-Fenton, among others, were not discussed. Nevertheless, researchers working on these types of topics in general should be advised that the Fenton process and its variants must be tested with actual wastewater on a large scale, since, over the past ten years, most research on the Fenton process and its variants has been conducted in synthetic solutions, which is far removed from reality [84] and means that these processes continue to present certain specific disadvantages, as shown in Table 6.

As final research directions, it is recommended that the toxicity of effluents generated by the Fenton process and its variants be studied. Evidence suggests that certain derivatives of emerging contaminants can make the effluent more toxic when this process is applied [87]. Likewise, research should focus on the competition between mineralization and by-product formation in the Fenton process. Finally, the Fenton process and its variants could be explored for transforming organic contaminants like emerging contaminants into polymers that can be separated from water. Polymerization systems driven by advanced oxidation processes, such as the Fenton process, are promising for reducing carbon emissions and achieving carbon recycling in water treatment [88].

### Other Reactions Involved in the Fenton Process

Other chemical reactions worth noting that occur in the Fenton process are Equations (15)–(18), shown below [89]:

(15) $$RH+HO^{•}\to R^{•}+H_{2}O$$

(16) $$R^{•}+H_{2}O_{2}\to ROH+HO^{•}$$

(17) $${Fe}^{3+}+HO_{2}^{•}\to {Fe}^{2+}+O_{2}+H^{+}$$

(18) $$R^{•}+O_{2}\to ROO^{•}$$

These reactions generate R^•^ and ROO^•^ radicals in the Fenton process, which may then form stable molecules or react with iron (Fe) ions, generating organic intermediates that can react with hydroxyl radicals (HO^•^) and oxygen (O_2_) to produce oxidation products and, in the best-case scenario, lead to mineralization by-products such as H_2_O, CO_2_, etc. [89].

## 5. Conclusions

The following conclusions can be drawn from the information gathered in this manuscript:

1. The homogeneous Fenton process and some of its variants (photo-Fenton and electro-Fenton) show the ability to remove emerging contaminants in different types of wastewater, either as single or coupled processes.
2. It is necessary to bring the application of the Fenton process and its variants imperatively to a macro scale.
3. It is time that the Fenton process and its variants are applied and tested for the removal of emerging contaminants mostly in real wastewater.
4. In the case of the Fenton-like and heterogeneous Fenton processes, it should be verified that the catalysts used and developed do not affect the environment (flora, fauna and human beings) and can be applied for the treatment of real wastewater at a macro level.

Finally, if another parameter is added to improve the traditional homogeneous Fenton process, as in the case of photo-Fenton or electro-Fenton, it must be verified that the addition does not complicate the process. Otherwise, while certain problems would be solved, more would be generated (Table 6), which would be counterproductive. That said, although the Fenton process was discovered in 1894, improvements are still needed for it to be applicable on a large scale for treating wastewater and removing emerging contaminants.

## Figures and Tables

**Table 1:** Variability of emerging contaminants in wastewater and their potential impacts on human health and the environment.

Group of Emerging Contaminants	Examples	Concentration Range	Main Environmental Impact	Main Human Health Impact	Reference
Pharmaceuticals	Ciprofloxacin, sulfamethoxazole, ibuprofen, diclofenac, acetaminophen	ng/L–10^5^ ng/L	Antimicrobial resistance, aquatic toxicity	Antibiotic resistance; microbiome disruption; liver, kidney, and gastrointestinal toxicity	[1,10]
Personal Care Products	Triclosan, triclocarban	ng/L–10^4^ ng/L	Aquatic toxicity, resistant bacteria	Endocrine and possible carcinogenic effects	
Hormones	Estradiol	ng/L–10^4^ ng/L	Hormonal disruption, reproductive toxicity in wildlife	Reproductive and developmental disorders	

**Table 2:** Examples of removal of emerging contaminants in different wastewater by the homogeneous Fenton process as a single or coupled treatment.

Emerging Contaminant(s)	Type of Wastewater	Operating Conditions	Treatment Time (Minutes)	% Removal	Reference
Benzene dye intermediates	Benzene dye production wastewater	pH = 4.13, [H_2_O_2_] = 1 M, [Fe^2+^] = 0.36 M	60	85.29%	[34]
Oil refinery compounds	Oil refinery wastewater	pH = 3, H_2_O_2_/COD = 2.8, H_2_O_2_/Fe^2+^ = 4 molar ratios	71	90% (calculated in function of COD)	[35]
Triclosan ^a^, ibuprofen ^a^, carbamazepine ^a^, caffeine ^a^, acesulfame-K ^a^ and DEET ^b^	Domestic wastewater from secondary process of a conventional WWTP	pH = 3, [H_2_O_2_] = [Fe^2+^] = not reported	60	100% ^a^, 85.21% ^b^	[31]
17α-ethinylestradiol ^a^ and caffeine ^b^	Wastewater from UASB	pH = 3, [Fe^2+^] = 0.5 mmolFe^2+^·L^−1^, 1 Fe^2+^: 10 H_2_O_2_ molar ratio	60	>99% ^a,b^	[36]

Note: The superscripts a and b appearing in the table, in the columns for emerging contaminants and % removal, refer to the removal rate obtained for each emerging contaminant. For example, DEET, which has a superscript b, had a removal rate of 85.21%, and the other compounds (triclosan, ibuprofen, etc.) in those columns that correspond to that row had a removal rate of nearly 100%.

**Table 3:** Examples of removal of emerging contaminants in different wastewater by the photo-Fenton process as a single or coupled treatment.

Emerging Contaminant(s)	Type of Wastewater	Operating Conditions	Treatment Time (Minutes)	% Removal	Reference
Acetamiprid	Synthetic secondary wastewater effluent	pH = 2.8, [Fe^2+^] = 1, 2, 3 mg-L^−1^. H_2_O_2_/Fe^2+^ = 2, 4 molar ratios, type of lamp = UVC	Not defined	70–90%	[48]
Epoxy paint compounds	Epoxy paint wastewater	pH = 3.5, H_2_O_2_/Fe^2+^ = 0.48 molar ratio, type of lamp = UVC multi lamp (38 W)	60	96.4% (calculated in function of COD)	[49]
Propanol	Secondary wastewater effluent spiked with propanol from membrane bioreactor ^a^ and integrated fixed-film activated sludge ^b^	pH = 2.8, [Fe^2+^] = 0.18 mmolFe^2+^·L^−1^, [H_2_O_2_] = 4.41 mmolH_2_O_2_·L^−1^, type of lamp = UV-A (8 W)	60	52.1% ^a^, 32.9% ^b^	[50]
Winery compounds	Winery wastewater	pH = 3, [FeSO_4_•7H_2_O] = 0.5 g·L^−1^, [H_2_O_2_] = 155 mmolH_2_O_2_·L^−1^, type of lamp = UVC	Not defined	98.9% (calculated in function of COD)	[51]

Note: The superscripts a and b refer to the % removal of the contaminant in each effluent from the processes listed in the “Type of Wastewater” column that were subjected to the photo-Fenton process.

**Table 4:** Examples of removal of emerging contaminants by the heterogeneous Fenton process.

Emerging Contaminant(s)	Type of Wastewater	Operating Conditions	Treatment Time (Minutes)	% Removal	Reference
Sulfamethoxazole	Synthetic	pH = 3, [H_2_O_2_] = 5 mmolH_2_O_2_·L^−1^, [Nanoscale Schwertmannite] = 0.1 g·L^−1^	90	92.5%	[65]
Bisphenol-A	Synthetic	pH = 6, $\left[LaCu_{0.5}Fe_{0.5}O_{3-\delta }\right]$ = 1 g·L^−1^, [H_2_O_2_] = 12 mmolH_2_O_2_-L^−1^	120	92.1%	[66]
Phenol	Synthetic	pH = 6.5, [CuNiFe layered double hydroxides] = 1 g·L^−1^, [H_2_O_2_]/[Phenol] = 37 molar ratio	60	98.9% (calculated in function of total organic carbon (TOC))	[67]

**Table 5:** Examples of removal of emerging contaminants in different wastewater by the electro-Fenton process.

Emerging Contaminant(s)	Type of Wastewater	Operating Conditions	Treatment Time (Minutes)	% Removal	Reference
Polymeric compounds	Pharmaceutical wastewater	pH = 3, [H_2_O_2_] 235.61 mg·L^−1^, [Fe^2+^] = 0.2 mmolFe^2+^·L^−1^, anode = boron-doped diamond, cathode = carbon brush, *j* = 4.17 mA·cm^−2^, air flow rate = 0.2 L·min^−1^	360	97% (calculated in function of total organic carbon (TOC))	[80]
Herbicide diquat dibromide	Contaminated water	pH = 2.2, [Fe^2+^] = 28 mg·L^−1^, *j* = 0.5 mA·cm^−2^, anode = cathode = boron-doped diamond	300	90% (calculated in function of total organic carbon (TOC))	[81]
Acid blue	Synthetic	pH = 3, anode = boron-doped diamond, cathode = carbon-PTFE screen, [catalyst] = iron mining waste = not reported, [H_2_O_2_] = not reported, air flow rate employed for electrogeneration H_2_O_2_ = 300 mL·min^−1^	20	100%	[82]

**Table 6:** Comparative overview of the Fenton process and its variants.

Variants of the Fenton Process	pH Operating Range	Catalyst Type	Oxidant	Energy Requirements	Advantages	Disadvantages
Homogeneous Fenton	Approximately 3 (acidity range)	Ferrous ion (Fe^2+^) as a homogeneous catalyst	H_2_O_2_	It does not require an external light source	Easy to operate, it is the basic Fenton process from which the other variants are derived	The generation of ferric sludge requires pH adjustment—both to lower it to acidic levels and to raise it to basic levels—which leads to higher reagent consumption
Photo-Fenton	Approximately 3 (acidity range)	Ferrous (Fe^2+^) or ferric (Fe^3+^) ions	H_2_O_2_	It requires an external light source	Three alternative routes to generate hydroxyl radicals (HO^•^)	If the wastewater is not adequately irradiated by the light source used, the removal of the target emerging contaminants may be reduced
Fenton-like	Approximately 3 (acidity range)	Fenton-like uses other metals (e.g., Cu^+^, Mn^2+^, and Co^2+^)	H_2_O_2_	It does not require an external light source	Use of different catalysts	It is necessary to assess the potential environmental impact of catalysts, their applicability at the macro level of treatment, their production costs, and their operating costs
Heterogeneous Fenton	Neutral pH ranges	Solid catalysts (e.g., Fe_2_O_3_, Fe_3_O_4_, FeO, FeOOH)	H_2_O_2_	It does not require an external light source	No ferric sludge is produced as a by-product, and no reagents are required for pH adjustment	Limitations in mass transfer and reaction kinetics and the lack of applicability of the heterogeneous Fenton process for treating actual wastewater at the macroscopic level
Electro-Fenton	Approximately 3 (acidity range)	See Section 3.4	H_2_O_2_	Electricity consumption	The oxidant agent (H_2_O_2_) is generated electrically in situ through the reduction of oxygen (O_2_) at the cathode	Long treatment retention times, scaling issues, electrode passivation, significant energy consumption, sludge generation (Fe(OH)_3_)

Note: Table 6 only lists the most significant advantages and disadvantages of the Fenton process and its variants. More information can be found throughout the document.

## Data Availability

No new data were created or analyzed in this study. Data sharing is not applicable to this article.

## References

[1] Parida V.K., Saidulu D., Majumder A., Srivastava A., Gupta B., Gupta A.K. (2021). Emerging contaminants in wastewater: A critical review on occurrence, existing legislations, risk assessment, and sustainable treatment alternatives. J. Environ. Chem. Eng..

[2] Reyes N.J.D.G., Geronimo F.K.F., Yano K.A.V., Guerra H.B., Kim L.-H. (2021). Pharmaceutical and Personal Care Products in Different Matrices: Occurrence, Pathways, and Treatment Processes. Water.

[3] Karpińska J., Kotowska U. (2021). New Aspects of Occurrence and Removal of Emerging Pollutants. Water.

[4] Mishra R.K., Mentha S.S., Misra Y., Dwivedi N. (2023). Emerging pollutants of severe environmental concern in water and wastewater: A comprehensive review on current developments and future research. Water-Energy Nexus.

[5] Bracamontes-Ruelas A.R., Ordaz-Díaz L.A., Bailón-Salas A.M., Ríos-Saucedo J.C., Reyes-Vidal Y., Reynoso-Cuevas L. (2022). Emerging Pollutants in Wastewater, Advanced Oxidation Processes as an Alternative Treatment and Perspectives. Processes.

[6] Polińska W., Kotowska U., Kiejza D., Karpińska J. (2021). Insights into the Use of Phytoremediation Processes for the Removal of Organic Micropollutants from Water and Wastewater; A Review. Water.

[7] Salamanca M., Peña M., Hernandez A., Prádanos P., Palacio L. (2023). Forward Osmosis Application for the Removal of Emerging Contaminants from Municipal Wastewater: A Review. Membranes.

[8] Mahmood T., Momin S., Ali R., Naeem A., Khan A., Ince M., Kaplan Ince O. (2022). Technologies for Removal of Emerging Contaminants from Wastewater. Wastewater Treatment.

[9] Bracamontes-Ruelas A.R., Irigoyen-Campuzano J.R., Torres-Castañon L.A., Reynoso-Cuevas L., Taşeli B.K. (2024). Application of Advanced Oxidation Processes for Domestic and Industrial Wastewater Treatment. Wastewater Treatment—Past and Future Perspectives.

[10] Jiang T., Wu W., Ma M., Hu Y., Li R. (2024). Occurrence and distribution of emerging contaminants in wastewater treatment plants: A globally review over the past two decades. Sci. Total Environ..

[11] Panghal P., Yousuf S., Sen S. (2024). Advanced oxidation processes and their application in the treatment of different types of wastewater samples. BIO Web Conf..

[12] Chen Y.-D., Duan X., Zhou X., Wang R., Wang S., Ren N.-Q., Ho S.-H. (2021). Advanced oxidation processes for water disinfection: Features, mechanisms and prospects. Chem. Eng. J..

[13] Umair M., Kanwal T., Loddo V., Palmisano L., Bellardita M. (2023). Review on Recent Advances in the Removal of Organic Drugs by Advanced Oxidation Processes. Catalysts.

[14] Ma D., Yi H., Lai C., Liu X., Huo X., An Z., Li L., Fu Y., Li B., Zhang M. (2021). Critical review of advanced oxidation processes in organic wastewater treatment. Chemosphere.

[15] Tao Y. (2022). Advanced Oxidation Processes for Water Purification Applications. Int. J. Innov. Entrep..

[16] Miklos D.B., Remy C., Jekel M., Linden K.G., Drewes J.E., Hübner U. (2018). Evaluation of advanced oxidation processes for water and wastewater treatment—A critical review. Water Res..

[17] Kaswan V., Kaur H. (2023). A comparative study of advanced oxidation processes for wastewater treatment. Water Pract. Technol..

[18] Pimentel Prates M., de Oliveira Loures Marcionílio S.M., Borges Machado K., Medeiros de Araújo D., Martínez-Huitle C.A., Leão Arantes A.L., Ferreira da Silva Gadêlha J.E. (2023). Fenton: A Systematic Review of Its Application in Wastewater Treatment. Processes.

[19] Jiang Y., Ran J., Mao K., Yang X., Zhong L., Yang C., Feng X., Zhang H. (2022). Recent progress in Fenton/Fenton-like reactions for the removal of antibiotics in aqueous environments. Ecotoxicol. Environ. Saf..

[20] Ameta R., Chohadia A.K., Jain A., Punjabi P.B., Ameta S.C., Ameta R. (2018). Fenton and Photo-Fenton Processes. Advanced Oxidation Processes for Waste Water Treatment.

[21] Fenton H.J.H. (1894). Oxidation of tartaric acid in the presence of iron. J. Chem. Soc. Trans..

[22] Salgado P., Frontela J.L., Vidal G. (2020). Optimization of Fenton Technology for Recalcitrant Compounds and Bacteria Inactivation. Catalysts.

[23] Babuponnusami A., Muthukumar K. (2014). A review on Fenton and improvements to the Fenton process for wastewater treatment. J. Environ. Chem. Eng..

[24] Thomas N., Dionysiou D.D., Pillai S.C. (2021). Heterogeneous Fenton catalysts: A review of recent advances. J. Hazard. Mater..

[25] Pignatello J.J., Oliveros E., MacKay A. (2006). Advanced Oxidation Processes for Organic Contaminant Destruction Based on the Fenton Reaction and Related Chemistry. Crit. Rev. Environ. Sci. Technol..

[26] Yin L., Wei J., Qi Y., Tu Z., Qu R., Yan C., Wang Z., Zhu F. (2022). Degradation of pentachlorophenol in peroxymonosulfate/heat system: Kinetics, mechanism, and theorical calculations. Chem. Eng. J..

[27] Chen J., Ma H., Luo H., Pu S. (2024). Mechanistic insights into the pH-driven radical transformation of the Fe (II)/nCP in groundwater remediation. J. Hazard. Mater..

[28] Zhang M.-H., Dong H., Zhao L., Wang D.-X., Meng D. (2019). A review on Fenton process for organic wastewater treatment based on optimization perspective. Sci. Total Environ..

[29] Bule Možar K., Miloloža M., Martinjak V., Radovanović-Perić F., Bafti A., Ujević Bošnjak M., Markić M., Bolanča T., Cvetnić M., Kučić Grgić D. (2024). Evaluation of Fenton, Photo-Fenton and Fenton-like Processes in Degradation of PE, PP, and PVC Microplastics. Water.

[30] Taco Ugsha M., Mayorga Llerena E. (2013). Aplicación del proceso Fenton en la disminución de materia orgánica en aguas residuales de la industria termoeléctrica. Quím. Cent..

[31] Bracamontes-Ruelas A.R., Reyes-Vidal Y., Irigoyen-Campuzano J.R., Reynoso-Cuevas L. (2023). Simultaneous Oxidation of Emerging Pollutants in Real Wastewater by the Advanced Fenton Oxidation Process. Catalysts.

[32] Chen Y., Cheng Y., Guan X., Liu Y., Nie J., Li C. (2019). A Rapid Fenton treatment of bio-treated dyeing and finishing wastewater at second-scale intervals: Kinetics by stopped-flow technique and application in a full-scale plant. Sci. Rep..

[33] Xu M., Wu C., Zhou Y., Bustillo-Lecompte C. (2020). Advancements in the Fenton Process for Wastewater Treatment. Advanced Oxidation Processes—Applications, Trends, and Prospects.

[34] Guo Y., Xue Q., Zhang H., Wang N., Chang S., Wang H., Pang H., Chen H. (2018). Treatment of real benzene dye intermediates wastewater by the Fenton method: Characteristics and multi-response optimization. RSC Adv..

[35] Ishak S., Malakahmad A. (2013). Optimization of Fenton process for refinery wastewater biodegradability augmentation. Korean J. Chem. Eng..

[36] López-Velázquez K., Villanueva-Rodríguez M., Mejía-González G., Herrera-López D. (2021). Removal of 17α-ethinylestradiol and caffeine from wastewater by UASB-Fenton coupled system. Environ. Technol..

[37] Huang Z., Huang X., Liu K., Fu J., Liu M. (2026). Degradation of per- and polyfluoroalkyl substances (PFASs) by Fenton reactions. Environ. Sci. Adv..

[38] Lin Y., Qiao J., Sun Y., Dong H. (2025). The profound review of Fenton process: What’s the next step?. J. Environ. Sci..

[39] Ribeiro J.P., Nunes M.I. (2021). Recent trends and developments in Fenton processes for industrial wastewater treatment—A critical review. Environ. Res..

[40] Mahtab M.S., Farooqi I.H., Khursheed A. (2021). Sustainable approaches to the Fenton process for wastewater treatment: A review. Mater. Today Proc..

[41] Kaviya S., Naushad M., Rajendran S., Lichtfouse E. (2020). Progression in Fenton Process for the Wastewater Treatment. Green Methods for Wastewater Treatment.

[42] Rueda-Márquez J.J., Levchuk I., Manzano M., Sillanpää M. (2020). Toxicity Reduction of Industrial and Municipal Wastewater by Advanced Oxidation Processes (Photo-Fenton, UVC/H_2_O_2_, Electro-Fenton and Galvanic Fenton): A Review. Catalysts.

[43] Rahim Pouran S., Abdul Aziz A.R., Wan Daud W.M.A. (2015). Review on the main advances in photo-Fenton oxidation system for recalcitrant wastewaters. J. End. Eng. Chem..

[44] Lucas M.S., Peres J.A. (2015). Removal of Emerging Contaminants by Fenton and UV-Driven Advanced Oxidation Processes. Water Air Soil Pollut..

[45] Sanabria P., Wilde M.L., Padillo A.R., Sirtori C. (2022). Trends in Fenton and photo-Fenton processes for degradation of antineoplastic agents in water matrices: Current knowledge and future challenges evaluation using a bibliometric and systematic analysis. Environ. Sci. Pollut. Res..

[46] Roslan N.N., Lau H.L.H., Suhaimi N.A.A., Shahri N.N.M., Verinda S.B., Nur M., Lim J.-W., Usman A. (2024). Recent Advances in Advanced Oxidation Processes for Degrading Pharmaceuticals in Wastewater—A Review. Catalysts.

[47] Prada-Vásquez M.A., Estrada-Flórez S.E., Serna-Galvis E.A., Torres-Palma R.A. (2021). Developments in the intensification of photo-Fenton and ozonation-based processes for the removal of contaminants of emerging concern in Ibero-American countries. Sci. Total Environ..

[48] Carra I., Sánchez Pérez J.A., Malato S., Autin O., Jefferson B., Jarvis P. (2015). Application of high intensity UVC-LED for the removal of acetamiprid with the photo-Fenton process. Chem. Eng. J..

[49] Balcioglu Ilhan E.B., Ilhan F., Kurt U., Yetilmezsoy K. (2024). An Optimization Study of Advanced Fenton Oxidation Methods (UV/Fenton–MW/Fenton) for Treatment of Real Epoxy Paint Wastewater. Water.

[50] (2020). 14th Mediterranean Congress of Chemical Engineering. https://intranet.pacifico-meetings.com/amsysweb/faces/publicacionOnlineDOI.xhtml?id=572&idComunicacion=140696.

[51] Jorge N., Teixeira A.R., Silva S., Pirra A., Peres J.A., Lucas M.S. (2023). Homogeneous vs. Heterogeneous Photo-Fenton Processes in the Treatment of Winery Wastewater. Eng. Proc..

[52] Han H., Li J., Santos H.A. (2023). Recent advances in Fenton and Fenton-like reaction mediated nanoparticle in cancer therapy. Biomed. Technol..

[53] Abe C., Miyazawa T., Miyazawa T. (2022). Current Use of Fenton Reaction in Drugs and Food. Molecules.

[54] Johnson M.B., Mehrvar M. (2022). Treatment of Actual Winery Wastewater by Fenton-like Process: Optimization to Improve Organic Removal, Reduce Inorganic Sludge Production and Enhance Co-Treatment at Municipal Wastewater Treatment Facilities. Water.

[55] Sarmento A.P., Borges A.C., Matos A.T.d., Romualdo L.L. (2020). Sulfamethoxazole and Trimethoprim Degradation by Fenton and Fenton-like Processes. Water.

[56] Shokri A., Fard M.S. (2022). A critical review in Fenton-like approach for the removal of pollutants in the aqueous environment. Environ. Chall..

[57] Wang N., Zheng T., Zhang G., Wang P. (2016). A review on Fenton-like processes for organic wastewater treatment. J. Environ. Chem. Eng..

[58] Hu Q., Yang T., Wang S., Xu L., Wu M., Yu D., Fu K., Luo J. (2025). Promoting •OH-dominant Fenton-like process over peracetic acid activation by ultrafine FeOx nanoclusters anchored carbonaceous nanosheets. Fundam. Res..

[59] Nerud F., Baldrian P., Gabriel J., Ogbeifun D. (2001). Decolorization of synthetic dyes by the Fenton reagent and the Cu/pyridine/H_2_O_2_ system. Chemosphere.

[60] Barreiro J.C., Capelato M.D., Martin-Neto L., Bruun Hansen H.C. (2007). Oxidative decomposition of atrazine by a Fenton-like reaction in a H_2_O_2_/ferrihydrite system. Water Res..

[61] Huang W., Brigante M., Wu F., Mousty C., Hanna K., Mailhot G. (2013). Assessment of the Fe(III)–EDDS Complex in Fenton-like Processes: From the Radical Formation to the Degradation of Bisphenol A. Environ. Sci. Technol..

[62] Rezaei F., Vione D. (2018). Effect of pH on Zero Valent Iron Performance in Heterogeneous Fenton and Fenton-like Processes: A Review. Molecules.

[63] Zhu Y., Zhu R., Xi Y., Zhu J., Zhu G., He H. (2019). Strategies for enhancing the heterogeneous Fenton catalytic reactivity: A review. Appl. Catal. B.

[64] Chen Y., Miller C.J., Waite D. (2021). Heterogeneous Fenton Chemistry Revisited: Mechanistic Insights from Ferrihydrite-Mediated Oxidation of Formate and Oxalate. Environ. Sci. Technol..

[65] Meng X., Wang L., Yang Y., Song Y., Yuan C. (2023). Heterogeneous Fenton-like Catalyzation of Nanoscale Schwertmannite for Sulfamethoxazole Degradation. Coatings.

[66] Pan K., Yang C., Hu J., Yang W., Liu B., Yang J., Liang S., Xiao K., Hou H. (2020). Oxygen vacancy mediated surface charge redistribution of Cu-substituted LaFeO_3_ for degradation of bisphenol A by efficient decomposition of H_2_O_2_. J. Hazard. Mater..

[67] Wang H., Jing M., Wu Y., Chen W., Ran Y. (2018). Effective degradation of phenol via Fenton reaction over CuNiFe layered double hydroxides. J. Hazard. Mater..

[68] Conde J.J., Abelleira S., Estévez S., González-Rodríguez J., Feijoo G., Moreira M.T. (2023). Improving the sustainability of heterogeneous Fenton-based methods for micropollutant abatement by electrochemical coupling. J. Hazard. Mater..

[69] Bello M.M., Raman A.A.A., Asghar A. (2019). A review on approaches for addressing the limitations of Fenton oxidation for recalcitrant wastewater treatment. Process. Saf. Environ. Prot..

[70] Yu X., Liu H., Huang Y., Li C., Kuang L., Zhong J., Zhu S., Gou Y., Wang Y., Zhang Y. (2023). A green edge-hosted zinc single-site heterogeneous catalyst for superior Fenton-like activity. Proc. Natl. Acad. Sci. USA.

[71] Tripathy D.B. (2025). Single-atom catalysts (SACs) and their sustainable applications. Inorg. Chem. Commun..

[72] Liu G., Luo D., Wang L., Wang C., Cao Y., Singh L., Ahmadzadeh S., He Z. (2023). Current status and future perspective in electro-Fenton techniques for wastewater treatment: A bibliometric review. Appl. Nanosci..

[73] Meijide J., Dunlop P.S.M., Pazos M., Sanromán M.A. (2021). Heterogeneous Electro-Fenton as “Green” Technology for Pharmaceutical Removal: A Review. Catalysts.

[74] Moratalla Á., Lacasa E., Cañizares P., Rodrigo M.A., Sáez C. (2022). Electro-Fenton-Based Technologies for Selectively Degrading Antibiotics in Aqueous Media. Catalysts.

[75] Rivera F.L., Menendez N., Mazarío E., Herrasti P. (2022). Electrofenton with Reticular Vitreous Carbon and Iron Oxide Nanoparticles for Dye Removal: A Preliminary Study. Appl. Sci..

[76] Shokri A., Nasernejad B., Sanavi Fard M. (2023). Challenges and Future Roadmaps in Heterogeneous Electro-Fenton Process for Wastewater Treatment. Water Air Soil Poll..

[77] Valencia-Valero L.C., Fajardo-Puerto E., Elmouwahidi A., Bailón-García E., Carrasco-Marín F., Pérez-Cadenas A.F. (2024). Facile Synthesis of Carbon-Based Inks to Develop Metal-Free ORR Electrocatalysts for Electro-Fenton Removal of Amoxicillin. Gels.

[78] Oturan N., Oturan M.A., Martínez-Huitle C.A., Rodrigo M.A., Scialdone O. (2018). Electro-Fenton Process: Background, New Developments, and Applications. Electrochemical Water and Wastewater Treatment.

[79] Santos M.C., Antonin V.S., Souza F.M., Aveiro L.R., Pinheiro V.S., Gentil T.C., Lima T.S., Moura J.P.C., Silva C.R., Lucchetti L.E.B. (2022). Decontamination of wastewater containing contaminants of emerging concern by electrooxidation and Fenton-based processes—A review on the relevance of materials and methods. Chemosphere.

[80] Olvera-Vargas H., Gore-Datar N., Garcia-Rodriguez O., Mutnuri S., Lefebvre O. (2021). Electro-Fenton treatment of real pharmaceutical wastewater paired with a BDD anode: Reaction mechanisms and respective contribution of homogeneous and heterogeneous •OH. Chem. Eng. J..

[81] Valenzuela A.L., Vasquez-Medrano R., Ibanez J.G., Frontana-Uribe B.A., Prato-Garcia D. (2017). Remediation of Diquat-Contaminated Water by Electrochemical Advanced Oxidation Processes Using Boron-Doped Diamond (BDD) Anodes. Water Air Soil Pollut..

[82] dos Santos A.J., da Costa Cunha G., Cruz D.R.S., Romão L.P.C., Martínez-Huitle C.A. (2019). Iron mining wastes collected from Mariana disaster: Reuse and application as catalyst in a heterogeneous electro-Fenton process. J. Electroanal. Chem..

[83] Cipriano Crapina L., Dzene L., Brendlé J., Fourcade F., Amrane A., Limousy L. (2021). Review: Clay-Modified Electrodes in Heterogeneous Electro-Fenton Process for Degradation of Organic Compounds: The Potential of Structural Fe(III) as Catalytic Sites. Materials.

[84] Raj R., Tripathi A., Das S., Ghangrekar M.M. (2024). Is waste-derived catalyst mediated electro-Fenton a sustainable option for mitigating emerging contaminants from wastewater?. Curr. Opin. Environ. Sci. Health.

[85] He H., Zhou Z. (2017). Electro-Fenton process for water and wastewater treatment. Crit. Rev. Environ. Sci. Technol..

[86] Nidheesh P.V., Trellu C., Vargas H.O., Mousset E., Ganiyu S.O., Oturan M.A. (2023). Electro-Fenton process in combination with other advanced oxidation processes: Challenges and opportunities. Curr. Opin. Electrochem..

[87] Pan X., Chen J., Wu N., Qi Y., Xu X., Ge J., Wang X., Li C., Qu R., Sharma V.K. (2018). Degradation of aqueous 2,4,40-Trihydroxybenzophenone by persulfate activated with nitrogen doped carbonaceous materials and the formation of dimer products. Water Res..

[88] Chen Y., Ren W., Ma T., Ren N., Wang S., Duan X. (2024). Transformative Removal of Aqueous Micropollutants into Polymeric Products by Advanced Oxidation Processes. Environ. Sci. Technol..

[89] Kastanek F., Spacilova M., Krystynik P., Dlaskova M., Solcova O. (2023). Fenton Reaction–Unique but Still Mysterious. Processes.

